# Overcoming resistance to belief revision and correction of misinformation beliefs: psychophysiological and behavioral effects of a counterfactual mindset

**DOI:** 10.1038/s41598-024-63230-5

**Published:** 2024-05-31

**Authors:** Jacob M. Rose, Odkhishig Ganbold, Anna M. Rose, Jay C. Thibodeau, Kristian Rotaru

**Affiliations:** 1 Department of Accounting, University of Northern Colorado, Greeley, CO 80639 USA; 2 Department of Medicine at Royal Melbourne Hospital, Melbourne Medical School, The University of Melbourne, Melbourne, VIC 3050 Australia; 3 Department of Accounting, Bentley University, Waltham, MA 02452 US; 4 Department of Accounting, Monash Business School, Monash University, Caulfield East, VIC 3145 Australia; 5 The Turner Institute for Brain and Mental Health, School of Psychological Sciences and Monash Biomedical Imaging Facility, Monash University, BrainPark, Clayton, VIC 3800 Australia

**Keywords:** Human behaviour, Psychology

## Abstract

In a series of experiments involving beliefs and misinformation beliefs, we find that individuals who are prompted with a counterfactual mindset are significantly more likely to change their existing beliefs when presented with evidence that contradicts their beliefs. While research finds that beliefs that are considered part of one’s identity are highly resistant to change in the face of evidence that challenges these beliefs, four experiments provide evidence that counterfactual generation causes individuals to adjust beliefs and correct misinformation beliefs in response to contradicting evidence. Indeed, we find that a counterfactual mindset was effective in promoting incorporation of accurate facts and causing individuals to revise misinformation beliefs about COVID vaccination safety for a large sample of individuals who have rejected COVID vaccinations. Finally, the results of the psychophysiological experiment reveal that counterfactual generation alters decision makers’ search strategies, increases their cognitive arousal in response to evidence that challenges their beliefs, and increases their desire to seek out disconfirming evidence. Overall, the four experiments indicate that counterfactual generation can effectively activate mindsets that increase individuals’ willingness to evaluate evidence that contradicts their beliefs and adjust their beliefs in response to evidence.

## Introduction

Misinformation beliefs and resistance to belief adjustment in response to quality evidence that contradicts these beliefs represent substantial challenges to society and are manifested in politics, public policy, health care, education, and nearly all aspects of life. When beliefs become part of one’s identity, these beliefs become highly resistant to change, even when substantial evidence directly contradicts these beliefs^1–6^. Increasing the capacity for evidence to influence beliefs and misinformation beliefs is critical for quality decision making, societal advance, and relevant public discourse. Research has investigated how misinformation beliefs form and how prebunking^7^ and debunking^8^ can help prevent the formation false beliefs (for a review, see Ecker and collaborators^9^). The current study takes a different perspective and investigates the potential for altering individuals’ mindsets to increase their receptiveness to evidence that challenges existing beliefs. In doing so, the study adopts an alternative approach to the one advocated by inoculation theory, which suggests that if people are exposed to weak versions of persuasive messages that challenge their existing beliefs or attitudes, they are better equipped to resist more persuasive messages that might attempt to change their views^10–12^. Thus, we examine how to improve the capacity for evidence to change beliefs that can be highly resistant to change. To accomplish this, the current study employs behavioral and psychophysiological experiments to investigate the potential for priming counterfactual mindsets to increase the capacity for evidence to influence beliefs and misinformation beliefs.

Counterfactual thinking is a reflection on what outcomes would, could, or should have been if events had happened differently^7,13–16^. Counterfactual generation is an everyday activity commonly reported by individuals in a variety of settings^13,14,17,18^. Research has demonstrated that counterfactual thinking during one task can prime decision makers to consider alternatives in subsequently encountered information, influencing their judgments in separate, unrelated tasks^19,20^. The effects of counterfactual thinking on subsequent judgments and decisions have been documented in the context of analytical problem-solving^15,19^, hypothesis-testing^19^, debiasing^21,22^, and information sharing in groups^23–25^.

For example, Galinsky and Moskowitz^19^ instructed subjects to read a story with a counterfactual scenario where a woman who was attending a rock concert wins (or loses) a trip to Hawaii immediately after switching her seat. The winning seat was the one that she had just moved to (or from). In a non-counterfactual scenario, the woman either wins or loses the trip, but the seat switching does not occur. After reading the story, subjects were asked to write down some of the thoughts that the woman might have had after the rock concert. The authors found that subjects exposed to a counterfactual scenario in the previous task were more sensitive to relevant alternative hypotheses. These results suggest that activating a counterfactual mindset in a context unrelated to a target task primes subjects in subsequent tasks to question initial hypotheses and attend to plausible alternative explanations in subsequently encountered information. These are precisely the types of decision processes that should increase the capacity of misinformation beliefs to be influenced by evidence that contradicts these beliefs.

We conduct four experiments to examine the effects of counterfactual generation on belief revision in the face of evidence that challenges existing beliefs. The first three experiments focus on the capacity for counterfactual generation in one task to affect the influence of factual information on existing beliefs, while the fourth experiment examines the effects of counterfactual generation on the capacity of facts to influence misinformation beliefs. The first two experiments examine whether a simple counterfactual generation task that is totally unrelated to beliefs about nuclear power can increase decision makers’ incorporation of new evidence into their existing beliefs about nuclear power. Results of the first two experiments reveal that counterfactual generation can effectively cause individuals to incorporate new evidence into their beliefs about nuclear power and alter their beliefs.

## Experiments one and two: beliefs about nuclear power

Two hundred twenty-three adult participants were recruited via Prolific for the first experiment, exceeding the minimum required sample size of 102 as determined by standard power analyses conducted with G*Power software. These analyses established the minimum acceptable sample size assuming medium effect sizes ($d = 0.5$) with a power of 0.8. There was a single data collection for all participants, and all analyses were conducted after collecting the full sample of participants. The 123 participants who held beliefs against nuclear power (i.e., scores of 1, 2 or 3) were included for the remainder of the experiment. The experiment was designed to test the capacity of counterfactual generation to increase the likelihood that pro-nuclear facts could change existing anti-nuclear beliefs. Therefore, participants needed to hold existing anti-nuclear beliefs. The participants first rated their beliefs about nuclear power support for nuclear power on a scale of 1 (not at all) to 7 (completely) and then read a short scenario about a war and were asked to list two ways that the outcome of the war could have turned out differently (counterfactual generation) or to list two facts about the war (no counterfactual generation). The story task is a previously validated task for activating a counterfactual mindset^26^. After reading the story and listing either two counterfactuals or two facts about the story, participants moved to the second phase. In the second phase, participants read a list of facts about nuclear power that contradicted their anti-nuclear beliefs. Finally, participants again rated their support for nuclear power. We tested changes in these beliefs against nuclear power in response to evidence that challenges existing beliefs. The second experiment was almost identical to the first, but we employed an alternative manipulation for the condition without counterfactual generation. 140 participants were recruited, with 108 holding anti-nuclear attitudes and completing the entire study. Instead of reading the story and listing two facts about the story, participants simply listed two random words.

## Results: experiments one and two

**Figure 1:**
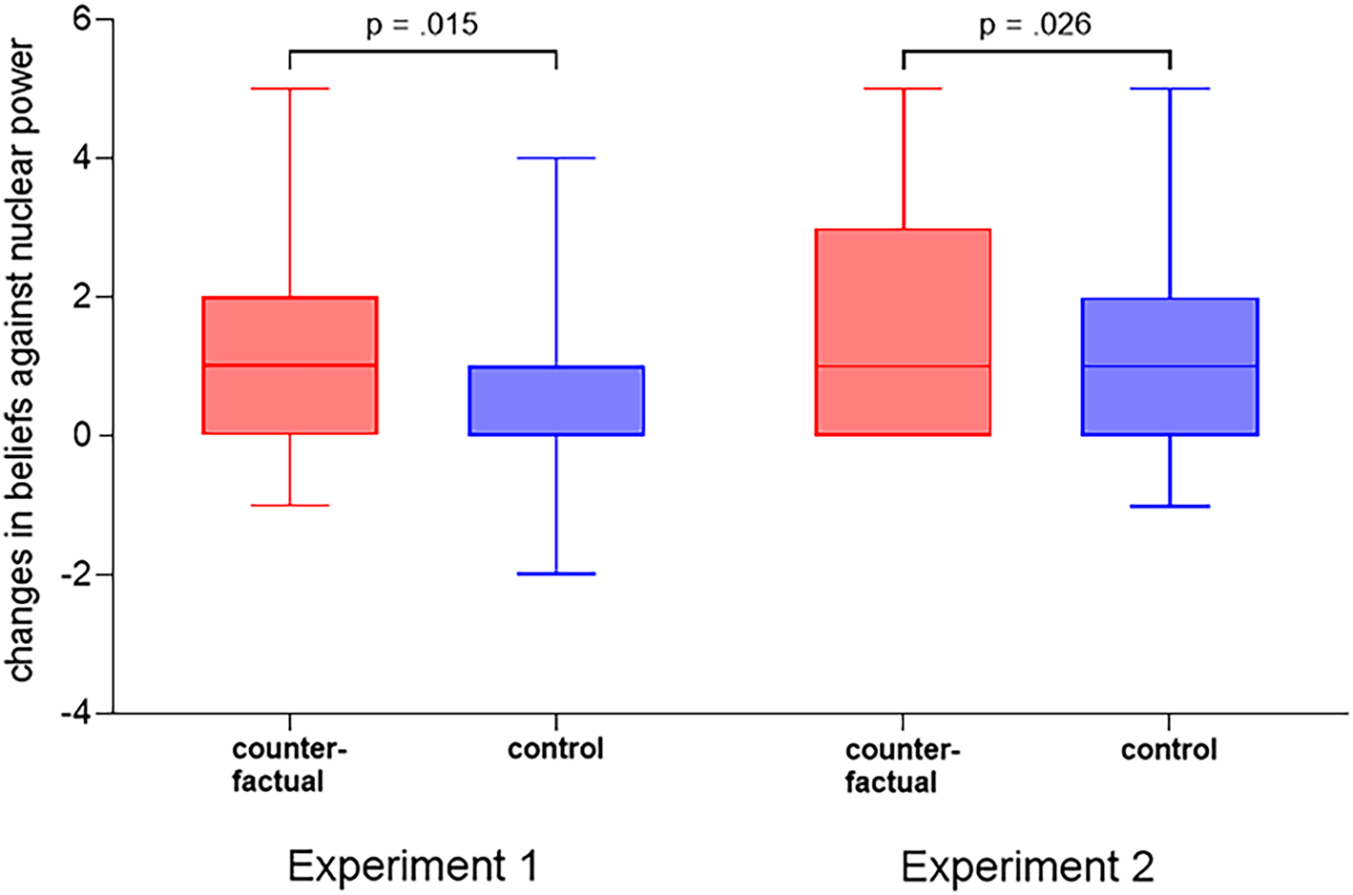
Boxplots: Changes in beliefs against nuclear power in response to evidence that challenges existing beliefs.

Behavioral results indicate that participants who generated counterfactuals before receiving evidence that challenged their beliefs about nuclear power changed their beliefs to be significantly more in favor of nuclear power (mean change = 1.18 in Experiment One and 1.64 in Experiment Two) than did participants who did not generate counterfactuals before receiving the evidence (mean change = 0.66 in Experiment One and 1.11 in Experiment Two). Based on independent samples t-tests, the changes are significantly different in Experiment One (see Fig. 1, $t(121) = 2.201$, $p = 0.015$, $d = 0.40$, one tailed) and Experiment Two (see Fig. 1, $t(106) = 1.959$, $p = 0.026$, $d = 0.38$, one tailed). The participants who generated counterfactuals increased their support for nuclear power 82% more in Experiment One and 48% more in Experiment Two after receiving evidence that contradicted anti-nuclear beliefs, relative to participants who had not generated counterfactuals.

## Experiment three: psychophysiological effects of counterfactual generation

The third experiment involves measurement of eye fixations and pupillary responses after completion of a counterfactual generation task. To consider plausible alternatives to existing beliefs, decision makers must attend to information that disconfirms existing beliefs and then encode and process this information. We investigate whether counterfactual generation causes decision makers to change their attention to and processing of disconfirming evidence by measuring psychophysiological responses to disconfirming evidence. Changes in attention can be readily measured with eye tracking technology, which makes it possible to precisely detect which information decision makers examine during their evaluation of evidence. Further, levels of encoding and processing of information are also revealed by psychophysiological responses, including pupillometry. Pupillometry measures changes in pupil diameter in response to various stimuli and change in pupil size has been regarded as a non-invasive proxy for neural activity^27^. Unsworth and Robinson (2017, p. 1964)^28^ refer to pupillary response “as an index of arousal and attentional effort”, thereby providing a non-invasive proxy measure of a decision maker’s arousal level^29–32^ and attentional effort^33–37^ in response to stimuli.

Arousal involves physiological and/or psychological responsiveness to internal or external stimuli^38^, and arousal levels reveal cognitive and emotional arousal and insight into effort levels, effort intensity, and attention intensity. Arousal influences individuals’ responses to stimuli, intensity of attention, and intensity of effort^33,38^, and arousal is essential for understanding and interpreting external stimuli^31,33,38,39^.

Pupillometry has been demonstrated to effectively measure cognitive and emotional arousal levels^34,40–48^. The capacity of pupil dilation to reveal arousal can be explained by the important role that the locus coeruleus noradrenergic arousal system (LC-NE), an important homeostatic control center of the body, plays in cognitive control as well as by the fact that pupil dilation is considered to be a common proxy for LC-NE firing^28,49,50^. In a recent study, Grueschow et al.^50^ showed that the LC-NE activity is functionally coupled to the activity in the dorso-medial prefrontal-cortex (DMPFC) and in the dorso-lateral prefrontal cortex (DLPFC), which provides further empirical evidence of the relationship between LC-NE, indexed by pupillary response, and top-down cognitive control. Finally, recent evidence demonstrates that the change in pupil dilation indicates the possible involvement of LC-NE neuromodulatory system in a psychological flow characterized by a full task immersion^30^.

By better understanding psychophysiological responses such as arousal and attention, we can study root cause effects of counterfactual generation on decision makers and advance theory that allows for more efficient and effective development of techniques to promote the consideration of evidence that contradicts existing beliefs. The third experiment involves assessing the effects of counterfactual generation on psychophysiological responses using pupillometry and eye tracking. Ninety-eight undergraduate students (32% female, 68% male) voluntarily participated in the study. All data were collected prior to any data analyses. Participant recruitment was guided via a student subject pool at a major Australian university. As an incentive, students received bonus credit in their course for participating in the experiment. The average age of the participants was 20.7 years ($SD = 1.48$). All participants had normal or corrected-to-normal vision.

The experimental design involves a single between-participant manipulation. The manipulation is the same counterfactual generation task that was employed in the first experiment. After reading the story and generating either two facts or two counterfactuals, participants completed an information search task. The search task comes from studies of information preference and choice and involves evaluation of recent news articles^51,52^. The news article task provides participants with the titles and summaries of recent news articles that either confirm or disconfirm their existing beliefs. Participants are provided with the titles and abstracts of six news articles related to nuclear power. Two articles favor nuclear power, two articles are against nuclear power, and two articles are neutral. The news article evaluations make it possible to determine whether generating counterfactuals in an unrelated task causes participants to be more likely to seek out and be more aroused by information that either confirms or disconfirms existing beliefs.

Three dependent variables used in this experiment represent individual psychophysiological responses while attending to different Areas of Interest (AOIs). The titles of the articles formed the AOIs, which were used to measure arousal and visual attention. We measured Pupillary Dilation, Fixation Duration, and Visit Count in relation to those AOIs. The fourth dependent variable captures participants’ desires to read the news articles that either confirm or disconfirm existing beliefs.

## Results: experiment three

**Figure 2:**
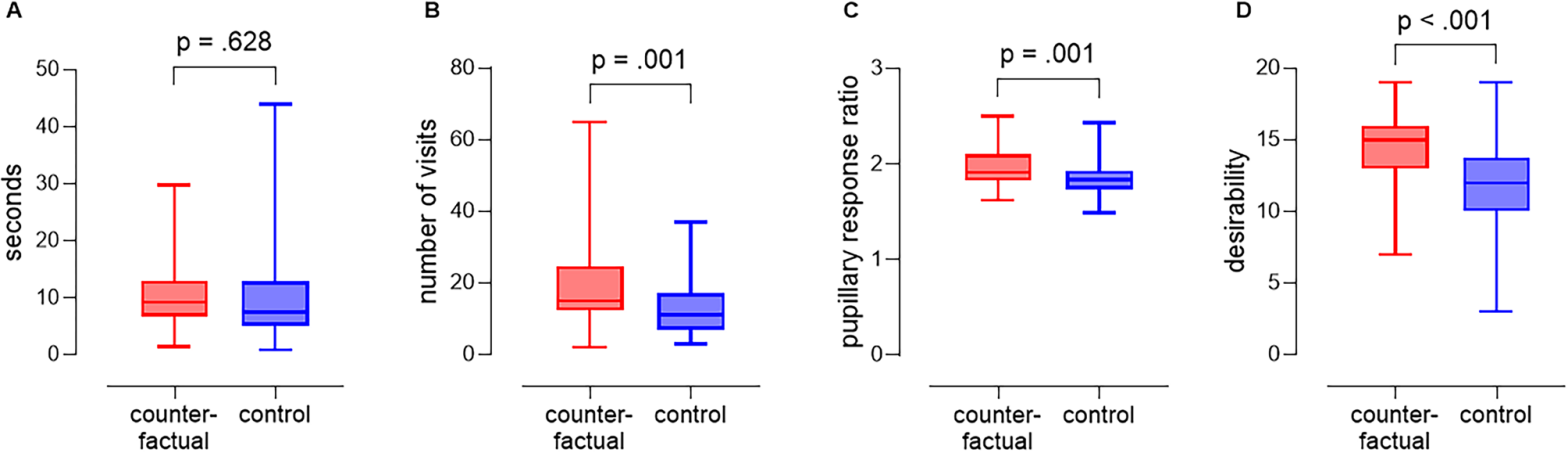
Boxplots: Psychophysiological and behavioral results. (**A**) Fixation duration. (**B**) AOI visit count. (**C**) Percentage change in pupil dilation. (**D**) Desirability to read disconfirming news articles.

We analyze eye tracking data to evaluate participants’ attention^53^. Based on an independent samples t-test, Fixation Duration does not differ across treatment conditions (see Fig. 2A, $t(91) = 0.486$, $p = 0.628$, two tailed), indicating no evidence of differences in the time duration of fixations upon reading titles that disconfirm their existing beliefs. An independent samples t-test Visit Count, however, indicates that participants who generated counterfactuals prior to the article viewing task attended more often to the titles that disconfirm their existing beliefs than did participants who had not generated counterfactuals (see Fig. 2B, $t(93) = 3.129$, $p = 0.001$, $d = 0.64$, one tailed). In addition to being statistically significant, the results are practically significant. Participants who generated counterfactuals exhibited approximately 50% more visits (mean visits = 19.3) to titles that disconfirmed their existing beliefs, relative to participants who did not generate counterfactuals in the prior task (mean visits = 12.8). Overall, counterfactual generation in a separate task completed before evaluation of evidence that conflicts with existing beliefs increases decision makers’ tendency to view information that disconfirms their existing beliefs.

Participants who generated counterfactuals in the task prior to examining evidence that conflicted with their beliefs also exhibited greater pupillary response (see Fig. 2C, $t(92) = 3.104$, $p = 0.001$, $d = 0.64$, one tailed) to titles that disconfirm their existing beliefs (mean = 1.98), relative to participants who did not generate counterfactuals (mean = 1.85). This result provides evidence that participants who generate counterfactuals are more aroused by disconfirming information, which can be indicative of increased processing and encoding of this information^54–56^. We further examine the effects of counterfactual generation on arousal associated with information that confirms existing beliefs. Participants who did not generate counterfactuals in a prior task exhibited greater pupillary response ($t(87.1) = -2.739$, $p = 0.004$, $d = -0.55$, one tailed) to titles that confirmed their existing beliefs, relative to participants who generated counterfactuals. The results indicate that participants who did not generate counterfactuals exhibited more processing and encoding of titles that helped to confirm existing beliefs, relative to participants who generated counterfactuals. The combination of increased propensity to attend to disconfirming evidence and increased arousal in response to disconfirming evidence suggest that substantial changes to decision making processes and information search strategies result from generating counterfactuals in unrelated tasks.

In addition to conducting the psychophysiological analyses, we also measured participants’ desires to read each of the news articles. For each article, participants indicated how desirable it would be to read each article on a scale of 1 (not at all desirable) to 11 (extremely desirable). Participants who had generated counterfactuals found the disconfirming stories to be significantly more desirable (see Fig. 2D, $t(96) = 4.823$, $p < 0.001$, $d = 0.98$, one tailed) than did participants who did not generate counterfactuals. The result is consistent with the psychophysiological results. In addition, participants who did not generate counterfactuals found the confirming stories to be significantly more desirable ($t(93.04) = -4.286$, $p < 0.001$, $d = -0.86$, one tailed) than did participants who did not generate counterfactuals.

Overall, results of the third experiment indicate that counterfactual generation in one task (i.e., generating counterfactuals from a war story) alters decision makers’ search strategies in an unrelated subsequent task (i.e., evaluating information that conflicts with existing beliefs about nuclear power), cognitive arousal in response to new information, and desire to seek out disconfirming evidence. Eye tracking results indicate that participants pay more attention to information that disconfirms existing beliefs after completion of a counterfactual generation task, relative to decision makers who do not complete a counterfactual generation task. Similarly, pupillometry results reveal increased pupillary response for information that disconfirms existing beliefs for decision makers who have generated counterfactuals, relative to decision makers who did not generate counterfactuals. These differences in pupillary response are indicative of increased cognitive and emotional arousal^34,40–48^, which are associated with increased attention, cognitive processing, and memory encoding^54–56^. Finally, participants also indicate greater preference for seeking out disconfirming information when they have generated counterfactuals in an unrelated task, relative to when they have not generated counterfactuals. Thus, the results provide evidence for the capacity of counterfactual generation to cause decision makers to seek out, process, and encode information that disconfirms their existing beliefs.

## Experiment four: misinformation beliefs about COVID vaccination safety

Given the promising findings from the first three experiments, the fourth experiment was designed to explore whether counterfactual generation could facilitate the revision of misinformation beliefs in response to contradictory evidence. A total of 591 adults from the United States and United Kingdom were recruited via Prolific, all of whom who have refused COVID vaccination. All data were collected in a single collection period in July 2022, and no analyses were completed until the data collection was complete. Among these, 560 adults who passed two attention checks were selected for data analysis. This number significantly exceeds the minimum required sample size of 102, as determined by standard power analyses conducted with G*Power software, which estimated the minimum acceptable sample size assuming medium effect sizes ($d = 0.5$) with a power of 0.8.

We evaluated changes in five common misinformation beliefs about COVID vaccination safety, including beliefs that the vaccination is dangerous, causes infertility, alters DNA, contributes to the creation of new variants, and provides less immunity than natural infection. The experimental manipulation involved the same counterfactual generation task from the second experiment. Participants first rated their beliefs about five false claims regarding COVID vaccination safety. Next, participants completed the counterfactual generation tasks where half listed two words and half indicated two ways the story could have ended differently. Next, participants read a list of facts that contradicted false claims about vaccine safety. Finally, participants again rated their beliefs about the false claims regarding vaccine safety.

## Results: experiment four

**Figure 3:**
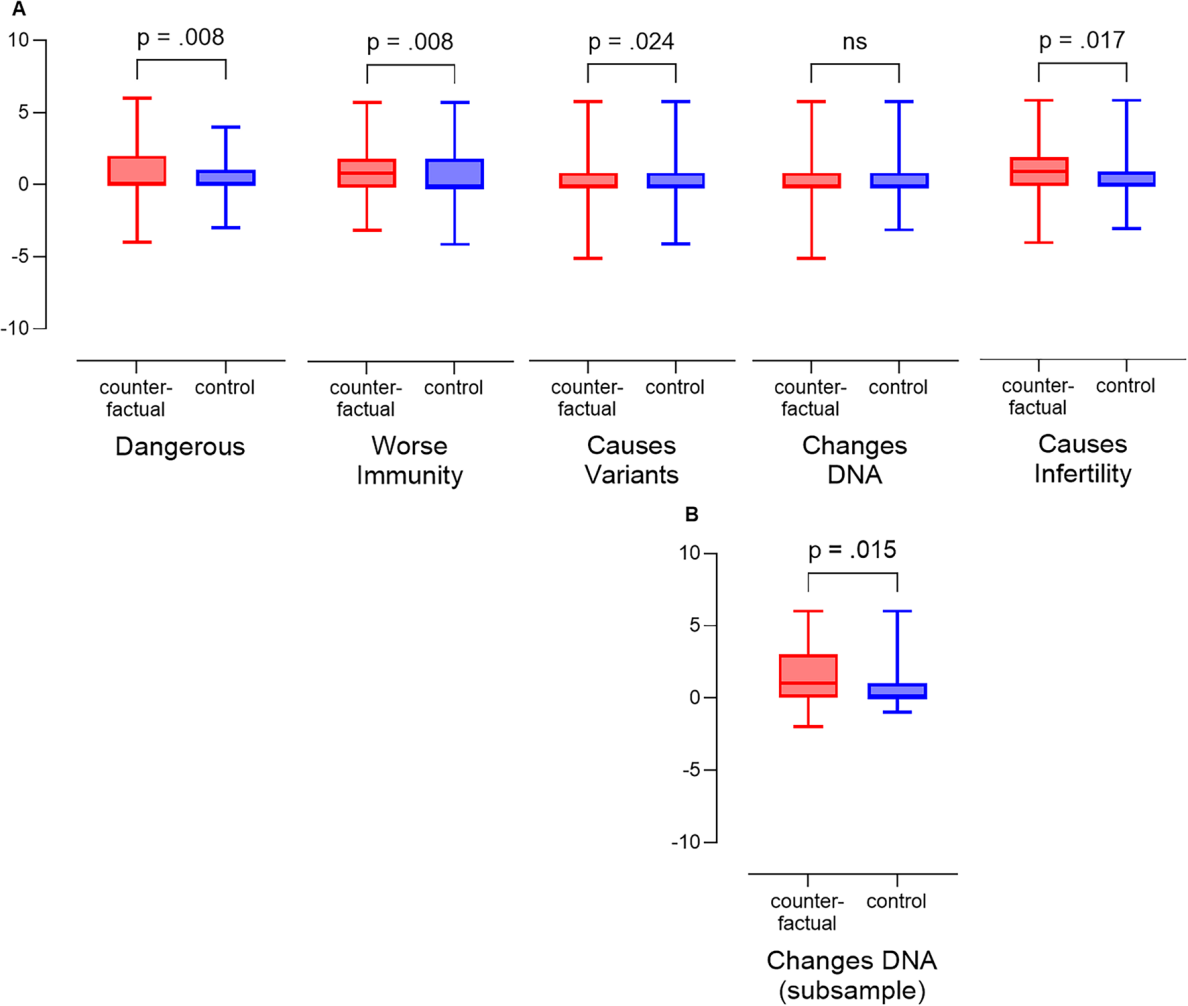
Boxplots: (**A**) Mean correction of COVID vaccination safety misbeliefs. (**B**) Mean correction of misbeliefs about DNA change—only participants who believed that DNA is changed.

Results of independent samples t-tests indicate that participants who generated counterfactuals before reading evidence that challenged false claims about vaccination safety made significantly larger adjustments to their misinformation beliefs about vaccination safety in response to the evidence. Based on an evaluation of all participants’ responses using a very conservative test with the least power to detect differences (i.e., a test that included participants who did not believe the false claim and therefore had limited or no opportunity to adjust their beliefs), counterfactual generation caused significantly greater adjustments in misinformation beliefs related to 4 of the false claims (dangerous, $t(558) = 2.409$, $p = 0.008$, $d = 0.20$; immunity, $t(548.52) = 2.433$, $p = 0.008$, $d = 0.21$; variants, $t(558) = 1.983$, $p = 0.024$, $d = 0.17$; fertility, $t(558) = 2.126$, $p = 0.017$, $d= 0.18$; all one tailed). See mean changes in Fig 3A. For the one false claim where there was no overall effect (i.e., false claim that vaccination causes DNA changes), we refine the test and examine only the 156 participants who believed the DNA misinformation before reading the contradictory evidence (i.e., participants who indicated a 5 or higher on the measurement scale). For these participants, the counterfactual caused a significant decrease in misinformation beliefs in response to the evidence (see Fig. 3B, $t(154) = 2.189$, $p = 0.015$, $d = 0.35$, one tailed). Thus, for all five common misinformation beliefs about COVID vaccination safety included in the study, the activation of a counterfactual mindset caused our participants to make more significant corrections to their misinformation beliefs. On average, the beliefs in misinformation were reduced 40% more for participants who generated counterfactuals, relative to those who did not, and results were consistent across participants from the U.S. and U.K. and across all levels of socioeconomic status.

## Discussion

We examined a method for increasing the capacity for facts and evidence to be successfully integrated into existing beliefs and misinformation beliefs. Our first two experiments demonstrated that generating counterfactuals before reviewing evidence that contradicts one’s beliefs increases belief adjustment in response to evidence. The third experiment then investigated the psychophysiological responses associated with counterfactual generation, such that we could better understand the root cause effects of counterfactual generation on belief revision. The results of eye tracking analyses indicated that participants attended more to information that disconfirmed their existing beliefs after completion of a counterfactual generation task. Further, participants exhibited increased pupillary response to information that disconfirmed their existing beliefs when they had generated counterfactuals, relative to participants who did not generate counterfactuals. Taken together, the psychophysiological measures suggest that counterfactual generation causes decision makers to seek out, process, and encode information that disconfirms their existing beliefs. Thus, activation of a counterfactual mindset appears to have substantial potential for opening people’s minds to evidence that counters misinformation beliefs.

The fourth experiment was designed to directly address the capacity of counterfactual generation to cause decreases in misinformation beliefs in the face of evidence that challenges misinformation beliefs. The results further support the potential benefits of counterfactual mindsets in the context of one of the world’s leading arenas of misinformation: COVID vaccination safety. Across a diverse sample of U.S. and U.K. participants who have refused vaccination, we find that counterfactual generation increases correction of misinformation beliefs in response to evidence that contradicts vaccine safety misinformation.

Ecker et al.^9^ discuss multiple methods that have been evaluated to prebunk and debunk misinformation and help prevent the formation of beliefs that are based on myths and false information. The current research evaluates a method to help overcome the effects of misinformation after it has caused false beliefs. Results from the four experiments suggest some exciting new pathways for more effectively disseminating facts that challenge misinformation. Activating counterfactual mindsets appears to open the mind to seeking, processing, and integrating evidence that challenges our belief and beliefs in misinformation. Methods that can help individuals correct misinformation beliefs have the capacity to reduce serious threats to society posed by the spread of misinformation. This research suggests several avenues for new research directions to help combat the effects of misinformation. For example, the current study is limited to examining the effects of counterfactual generation on consideration of evidence that challenges existing beliefs and is evaluated shortly after the act of generating counterfactuals. Additional research could examine whether other behavioral interventions have the capacity to create longer-term effects that open the mind to evidence conflicting with existing beliefs for longer periods of time. We also cannot speak to the duration of changes to misinformation beliefs. There is an opportunity to examine the persistence of changes to misinformation beliefs and the effects that such changes have on behavior and decision making. There will be significant value in understanding the potential long-term benefits to society of interventions that make individuals more receptive to evidence that challenges their misinformation beliefs.

## Methods

All experiments were approved by Monash University’s Human Research Ethics Committee, all experiments followed the relevant guidelines and regulations, and informed consent was obtained.

### Experiments one and two

These studies employ an existing counterfactual generation task that has previously been demonstrated to be effective for inducing an alternative-generation mindset^26^. The participants read a short story about the war between the British and the Gurkhas of Nepal during the British consolidation of power in India. In the counterfactual generation condition, participants were instructed to list two thoughts about how the outcome of the story could have turned out differently. In the two types of conditions without counterfactual generation, participants were asked to either list two facts about the story or to list two random words.

### Experiment three

The first two dependent variables reflect characteristics of participants’ visual attention and take into consideration either the number or duration of their fixations on the AOIs. The first DV, Fixation Duration, is measured using eye tracking technology and represents the sum of the duration for all visual fixations within a target AOI^57^. A fixation occurs when the eyes stay focused on a single location on the screen of the eye tracking device (used for presenting all experimental materials) longer than a particular time threshold^57^. The Identification by Velocity - Threshold (IV-T) event detection algorithm^58,59^ was adopted via the selection of global settings in the eye tracking software (Tobii Studio 3.4.5). The default threshold of 60ms was selected within IV-T Tobii filter parameters to define fixations. Thus, a fixation is recorded when participants fixate at least 60ms on a particular spot on the screen.

The second dependent variable, Visit Count, is another eye tracking measure, which denotes the number of ‘visits’ specific to a target AOI. According to the accepted classification of the eye gaze data, a ‘visit is defined as the portion of gaze data between the start of the first fixation on the AOI until the end of the last fixation on the AOI, before the attention of the individual is fixated outside the target AOI^57^. Reflecting the number of ‘returns’ to the target AOI, this measure allows us to capture the intent of the participant to revisit the information presented within the target AOI.

We examine the duration of fixations and the number of visits to titles and abstracts of news stories (considered as target AOIs) that disconfirm or confirm existing beliefs. In order to categorize articles as either confirming or disconfirming existing beliefs for these analyses, we asked participants to indicate their beliefs about nuclear power prior to completing the news article task. Participant indicated their support for nuclear energy on a scale of 0 (Not at All) to 7 (Completely). We then categorized each individual as for or against nuclear power based on the median value of the measurement scale, i.e., splitting into those who are against nuclear power (0–3) and those for nuclear power (4–7).

The third dependent variable, Pupillary Response Ratio, is a continuous proportional measure, calculated using divisive approach to baseline correction^60,61^. Namely, it reflects a proportional difference from individual’s baseline pupil size. Using an individual’s mean response while attending to all AOIs as a baseline provides a conservative and highly reliable measure of change associated with attending to each individual AOI^32^. This approach also makes it possible to control for individual differences in pupil size. In recent reviews of the experimental pupillometry literature, increased proportional pupillary response is associated with higher levels of cognitive and emotional arousal, as well as attentional effort^31,32,37^. Using this measure, the AOI that results in a larger increase in proportional pupillary response is the AOI that produces higher levels of arousal.

Eye movements were recorded using a table-mounted eye tracking system (Tobii TX300) with a temporal resolution of 300 Hertz (Hz) and a screen resolution of $1920 \times 1080$ pixels (see Appendix for a more complete overview of technical specifications of the eye tracking measurements conducted in this study, which will be useful for future replication). The average viewing distance was 65 cm from the screen (range: 50–80 cm), binocular accuracy was 0.4$\circ$ and precision 0.14$\circ$. Fixations were computed using the velocity-based I-VT algorithm^58^. To define fixations, a conservative 60ms threshold was selected, denoting that any fixation below 60ms threshold was considered a saccade (i.e., a rapid movement of the eye between fixation points) and was not included in the analysis.

The experiment was conducted in a light-controlled, dimly lit booth. Participants sat on height-adjustable chairs with their head supported by a height-adjustable forehead and chin rest (Heavy Duty Chin Rest with Clamps from Richmond Products, Inc.). At the beginning of the experiment, the eye tracker was calibrated using a nine-point fixation technique, which is the most rigorous calibration technique for the device used. This calibration adjusts for participants’ individual differences in eye characteristics and their seating position.

### Experiment four

The 560 participants for the third experiment were recruited on Prolific Academic online research platform. The mean age of participants was 37.4 years, 53.4% identified as female, and 63% were from the United States (while the rest of the participants were from the United Kingdom). Participants were pre-screened to include only those who had refused any COVID vaccinations throughout the pandemic.

### Supplementary Information

Supplementary Information 1.

Supplementary Information 2.

## Data Availability

De-identified data are available from the corresponding author upon request.
