# Supplementary material for: Overcoming resistance to belief revision and correction of misinformation beliefs: psychophysiological and behavioral effects of a counterfactual mindset
Source: Sci Rep. 2024 May 31;14:12493. doi: 10.1038/s41598-024-63230-5 (PMC11143297; doi:10.1038/s41598-024-63230-5)
Supplement: Supplementary file 1 — Supplementary Information 1. [file 41598_2024_63230_MOESM1_ESM.docx]

## **Table S1**. Experiment 3 - Independent Samples T-Test and Descriptive Statistics for *Fixation Duration, Visit Count, and Pupillary Response Ratio* Across Treatment Groups with Disconfirming and/or Confirming Titles and Summaries

| **Area of Interest (AOI) type** | **measures** | **content type (with respect to belief)** | **treatment groups** | **obser-**  **vation** | **mean** | **std.err.** | **std.dev.** | **df** | **t-statistic** | **p-value  (one-tailed)** | **[95% conf. interval]** | | **cohen's d** |
| --- | --- | --- | --- | --- | --- | --- | --- | --- | --- | --- | --- | --- | --- |
| **Title** | *fixation duration* | *disconfirming* | *counterfactual* | 42 | 10.96 | 1.03 | 6.67 | 91 | 0.486 | *0.314* | -2.455 | 4.046 | 0.10 |
|  |  |  | *control* | 51 | 10.16 | 1.22 | 8.70 |  |  |  |  |  |  |
|  |  | *confirming* | *counterfactual* | 43 | 9.42 | 0.90 | 5.88 | 90 | -1.122 | *0.868* | -4.927 | 1.370 | -0.23 |
|  |  |  | *control* | 49 | 11.20 | 1.26 | 8.81 |  |  |  |  |  |  |
|  | *visit count* | *disconfirming* | *counterfactual* | 44 | 19.27 | 1.84 | 12.20 | 71.15 | 3.033 | *0.002* | 2.223 | 10.754 | 0.64 |
|  |  |  | *control* | 51 | 12.78 | 1.09 | 7.81 |  |  |  |  |  |  |
|  |  | *confirming* | *counterfactual* | 44 | 16.32 | 1.54 | 10.23 | 91 | 1.309 | *0.097* | -1.264 | 6.145 | 0.27 |
|  |  |  | *control* | 49 | 13.88 | 1.10 | 7.69 |  |  |  |  |  |  |
|  | *pupillary response ratio* | *disconfirming* | *counterfactual* | 42 | 1.98 | 0.03 | 0.22 | 92 | 3.104 | *0.001* | 0.047 | 0.216 | 0.64 |
|  |  |  | *control* | 52 | 1.85 | 0.03 | 0.19 |  |  |  |  |  |  |
|  |  | *confirming* | *counterfactual* | 43 | 1.84 | 0.02 | 0.16 | 87.1 | -2.739 | *0.004* | -0.191 | -0.030 | -0.55 |
|  |  |  | *control* | 50 | 1.95 | 0.03 | 0.23 |  |  |  |  |  |  |
| **Summary** | *fixation duration* | *disconfirming* | *counterfactual* | 45 | 28.57 | 2.85 | 19.11 | 94 | 0.286 | *0.388* | -6.484 | 8.666 | 0.06 |
|  |  |  | *control* | 51 | 27.48 | 2.55 | 18.24 |  |  |  |  |  |  |
|  |  | *confirming* | *counterfactual* | 45 | 27.25 | 2.21 | 14.81 | 94 | 0.185 | *0.427* | -5.402 | 6.510 | 0.04 |
|  |  |  | *control* | 51 | 26.70 | 2.04 | 14.54 |  |  |  |  |  |  |
|  | *visit count* | *disconfirming* | *counterfactual* | 45 | 21.98 | 2.63 | 17.65 | 68.54 | 2.141 | *0.018* | 0.436 | 12.382 | 0.45 |
|  |  |  | *control* | 51 | 15.57 | 1.43 | 10.20 |  |  |  |  |  |  |
|  |  | *confirming* | *counterfactual* | 45 | 20.96 | 2.21 | 14.82 | 67.7 | 2.763 | *0.004* | 1.921 | 11.912 | 0.58 |
|  |  |  | *control* | 51 | 14.04 | 1.18 | 8.40 |  |  |  |  |  |  |
|  | *pupillary response ratio* | *disconfirming* | *counterfactual* | 45 | 1.95 | 0.03 | 0.19 | 95 | 1.926 | *0.029* | -0.003 | 0.166 | 0.39 |
|  |  |  | *control* | 52 | 1.87 | 0.03 | 0.22 |  |  |  |  |  |  |
|  |  | *confirming* | *counterfactual* | 45 | 1.84 | 0.02 | 0.14 | 86.51 | -2.932 | *0.002* | -0.180 | -0.034 | -0.58 |
|  |  |  | *control* | 52 | 1.95 | 0.03 | 0.22 |  |  |  |  |  |  |

## **Table S2**. Technical specifications of oculomotor and pupillometry measurements

| **Core parameters** | **Parameter description** | **Parameter specifications adopted in the experiment** |
| --- | --- | --- |
| **Eye Tracking Measures**  **Used** | (Total) Fixation Duration | The aggregate length of time an individual’s gaze is fixed on a specific Area of Interest (AOI) |
|  | Visit count | The total number of distinct time intervals during which an individual’s gaze is focused on a specific Area of Interest (AOI) from the initiation of the first fixation to the conclusion of the last fixation within that AOI. |
|  | Pupillary Response Ratio | A continuous proportional metric that calculates the percentage change in pupil size from a baseline diameter when a person responds to an AOI either through dilation or constriction. |
| **Apparatus** | Sampling procedure | Binocular recording procedure was used (i.e. pupil dilation and eye tracking measures are based on the data acquired from both left and right eyes of the participants) |
|  | Name and produce of the eye tracking device | Tobii TX300, Tobii (Sweden) |
|  | Type of eye tracking device | Desk-mounted |
|  | Sampling rate | 300 Hz |
|  | Sampling rate variability | 0.3% |
|  | Processing latency | 1.0 – 3.3 ms |
|  | Accuracy (the angular average distance from the actual gaze point to the one measured by the eye tracker) | 0.4^0^ – at ideal conditions, 0.3^0^ - at 25^0^ gaze, 0.6^0^ - at 30^0^ gaze, 0.6^0^ – at 1 lux, 0.4^0^ – at 300 lux, 0.5^0^ – at 600 lux, 0.5^0^ – at 1000 lux. |
|  | Precision | 0.01^0^ – with Stamper filter (for more details on the applied Stampe algorithm for noise reduction see Stampe (1993)) |
|  | Eye tracking software used | Tobii Studio 3.4.5 |
|  | Chin rest used | Yes |
| **Monitor** | Screen size | 23ˈ̎ |
|  | Screen resolution | 1920 x 1080 pixel |
|  | Distance between participant and screen | Operating distance: 50-80cm  Default distance used in this study: 65cm |
| **Calibration** | How many points in calibration | 9-point calibration |
|  | Amount of recalibration | No recalibration used |
| **Materials/Areas of Interest (AOIs)** | AOIs used for eye tracking data analysis | Article titles and summary information (presented in Appendix A) |
| **Participant characteristics** | Participant vision (corrected or not) | Normal and corrected-to-normal vision. |
| **Event detection** | What algorithm is used for event detection | The Identification by Velocity - Threshold (IV-T) event detection algorithm (Komogortsev et al., 2010; Andersson et al., 2017) was adopted via the selection of global settings in the eye tracking software (Tobii Studio 3.4.5). The default threshold of 60ms was selected within IV-T Tobii filter parameters to define fixations. |

**References**

Andersson, R., Larsson, L., Holmqvist, K., Stridh, M., & Nyström, M. (2017). One algorithm to rule them all? An evaluation and discussion of ten eye movement event-detection algorithms. *Behavior Research Methods, 49*, 616-637.

Komogortsev, O. V., Gobert, D. V., Jayarathna, S., & Gowda, S. M. (2010). Standardization of automated analyses of oculomotor fixation and saccadic behaviors. *IEEE Transactions on Biomedical Engineering, 57*(11), 2635-2645.

Stampe, D. M. (1993). Heuristic filtering and reliable calibration methods for video-based pupil-tracking systems. *Behavior Research Methods, Instruments, & Computers*, 25(2), 137-142.

## **APPENDIX A.** Research instruments

**EXPLANATORY STATEMENT** *(All experiments began with an ethics approved explanatory statement and consent. )*

## EXPERIMENT ONE

PART 1. Test of existing belief *(randomly presented with a filler question)*

Do you support the use of nuclear energy?

| 1  Not at all | 2 | 3 | 4 | 5 | 6 | 7  Completely |
| --- | --- | --- | --- | --- | --- | --- |

Please indicate your level of agreement with the following statement:
At any given time, humans only use about 10% of their brain's capacity. *(filler question)*

| 1  Strongly disagree | 2 | 3 | 4 | 5 | 6 | 7  Strongly agree |
| --- | --- | --- | --- | --- | --- | --- |

### PART 2. Experimental manipulation

1. **Counterfactual generation condition:**

Please read the following story and then answer the question.

For some years after the arrival of Hastings as Great Britain’s Governor-General of India, the consolidation of power involved serious war. The first of these wars took place on the northern frontier of Bengal where the British were faced by plundering raids of the Gurkhas of Nepal. Attempts had been made to stop raids by an exchange of lands, but the Gurkhas would not give up their claims to a country under British control, and Hastings decided to deal with them once and for all. The campaign began in November, 1814. It was not glorious. The Gurkhas were only some 12,000 strong; but they were brave fighters, fighting in a territory well-suited to their raiding tactics. The older British commanders were used to war in the plains where the enemy would typically run away from a resolute attack. In the mountains of Nepal it was difficult to even find the enemy. The troops and transport animals suffered from the extremes of heat and cold, and the officers were slow to learn caution. The British overcame the Gurkhas and ultimately won the war.

Please list 2 thoughts about how this scenario might have turned out differently. That is, how could the Gurkhas have won this war?

1. __________________________________________________________
2. _________________________________________________________
3. **Control condition:**

Please read the following story and then answer the question.

For some years after the arrival of Hastings as Great Britain’s Governor-General of India, the consolidation of power involved serious war. The first of these wars took place on the northern frontier of Bengal where the British were faced by plundering raids of the Gurkhas of Nepal. Attempts had been made to stop raids by an exchange of lands, but the Gurkhas would not give up their claims to a country under British control, and Hastings decided to deal with them once and for all. The campaign began in November, 1814. It was not glorious. The Gurkhas were only some 12,000 strong; but they were brave fighters, fighting in a territory well-suited to their raiding tactics. The older British commanders were used to war in the plains where the enemy would typically run away from a resolute attack. In the mountains of Nepal it was difficult to even find the enemy. The troops and transport animals suffered from the extremes of heat and cold, and the officers were slow to learn caution. The British overcame the Gurkhas and ultimately won the war.

Please list 2 facts about this scenario.

1. __________________________________________________________
2. __________________________________________________________

PART 3. Pro-nuclear facts *(randomly presented)*

On the following pages, you will be provided with information that was collected from reputable sources. It is important that you read all of the information because you will be asked to consider this information in the next section.

***Findings related to Nuclear Power:***

- We need more carbon-free power, not less. By nearly all accounts, nuclear energy is the most rapidly scalable form of carbon-free power invented. And the technology is rapidly improving. Nuclear power plants emit no carbon pollution, no carbon monoxide, no sulphur oxides and no nitrogen oxides to the atmosphere.
- Nuclear energy provides a sustainable and safer supply at lower costs, addressing vulnerabilities to disruptions in oil and gas supplies. It also ensures energy security with stable costs, reducing the impact of fuel price fluctuations and offering cheaper electricity to consumers compared to fossil fuels.
- The world needs nuclear energy for electric vehicles. Global demand for electricity is predicted to grow 76% by 2030, and nuclear energy is set to play a key role in meeting the demand. Even a modest increase in the penetration of electric vehicles into the automotive market will require a significant increase in the development of nuclear energy.
- Nuclear energy is used to power deep space missions. Radioisotope thermoelectric generators (RTGs) have been used to power spacecraft. These devices directly convert heat from decaying plutonium into electricity. The devices have no moving parts, making them ideal for applications in space.

*(Attention check)*

***Findings related to Brain Capacity:***

- All neurons have a resting firing rate, therefore they are never completely idle or unused.
- There is no part of the brain which is not active over the course of day.
- The process of natural selection generally does not yield biological adaptations that are not useful.
- The myth that humans use 10% of their brains is not supported by any neuroscientific evidence.

PART 4. Retest of belief *(randomly presented)*

Do you support the use of nuclear energy?

| 1  Not at all | 2 | 3 | 4 | 5 | 6 | 7  Completely |
| --- | --- | --- | --- | --- | --- | --- |

Please indicate your level of agreement with the following statement:
At any given time, humans only use about 10% of their brain's capacity. *(filler question)*

| 1  Strongly disagree | 2 | 3 | 4 | 5 | 6 | 7  Strongly agree |
| --- | --- | --- | --- | --- | --- | --- |

### PART 5. Demographics questions

What is your age? _________

How would you describe your gender?

- Male
- Female
- Non-binary / non-conforming
- Other (please specify) ______________
- Prefer not to say

## EXPERIMENT TWO

PART 1. Test of existing belief *(randomly presented with a filler question)*

Do you support the use of nuclear energy?

| 1  Not at all | 2 | 3 | 4 | 5 | 6 | 7  Completely |
| --- | --- | --- | --- | --- | --- | --- |

Please indicate your level of agreement with the following statement:
At any given time, humans only use about 10% of their brain's capacity. *(filler question)*

| 1  Strongly disagree | 2 | 3 | 4 | 5 | 6 | 7  Strongly agree |
| --- | --- | --- | --- | --- | --- | --- |

### PART 2. Experimental manipulation

1. **Counterfactual generation condition:**

Please read the following story and then answer the question.

For some years after the arrival of Hastings as Great Britain’s Governor-General of India, the consolidation of power involved serious war. The first of these wars took place on the northern frontier of Bengal where the British were faced by plundering raids of the Gurkhas of Nepal. Attempts had been made to stop raids by an exchange of lands, but the Gurkhas would not give up their claims to a country under British control, and Hastings decided to deal with them once and for all. The campaign began in November, 1814. It was not glorious. The Gurkhas were only some 12,000 strong; but they were brave fighters, fighting in a territory well-suited to their raiding tactics. The older British commanders were used to war in the plains where the enemy would typically run away from a resolute attack. In the mountains of Nepal it was difficult to even find the enemy. The troops and transport animals suffered from the extremes of heat and cold, and the officers were slow to learn caution. The British overcame the Gurkhas and ultimately won the war.

Please list 2 thoughts about how this scenario might have turned out differently. That is, how could the Gurkhas have won this war?

1. __________________________________________________________
2. __________________________________________________________
3. **Control condition:**

Please list two random words.

1. __________________________________________________________
2. __________________________________________________________

PART 3. Pro-nuclear facts *(randomly presented)*

On the following pages, you will be provided with information that was collected from reputable sources. It is important that you read all of the information because you will be asked to consider this information in the next section.

***Findings related to Nuclear Power:***

- We need more carbon-free power, not less. By nearly all accounts, nuclear energy is the most rapidly scalable form of carbon-free power invented. And, the technology is rapidly improving. Nuclear power plants emit no carbon pollution, no carbon monoxide, no sulphur oxides and no nitrogen oxides to the atmosphere.
- Nuclear energy is used to power deep space missions. Radioisotope thermoelectric generators (RTGs) have been used to power spacecraft. These devices directly convert heat from decaying plutonium into electricity. The devices have no moving parts, making them ideal for applications in space.
- The world needs nuclear energy for electric vehicles. Global demand for electricity is predicted to grow 76% by 2030, and nuclear energy is set to play a key role in meeting the demand. Even a modest increase in the penetration of electric vehicles into the automotive market will require a significant increase in the development of nuclear energy.

*(Attention check)*

***Findings related to Brain Capacity:***

- All neurons have a resting firing rate, therefore they are never completely idle or unused.
- There is no part of the brain which is not active over the course of day.
- The process of natural selection generally does not yield biological adaptations that are not useful.
- The myth that humans use 10% of their brains is not supported by any neuroscientific evidence.

PART 4. Retest of belief *(randomly presented with a filler question)*

Do you support the use of nuclear energy?

| 1  Not at all | 2 | 3 | 4 | 5 | 6 | 7  Completely |
| --- | --- | --- | --- | --- | --- | --- |

Please indicate your level of agreement with the following statement:
At any given time, humans only use about 10% of their brain's capacity. *(filler question)*

| 1  Strongly disagree | 2 | 3 | 4 | 5 | 6 | 7  Strongly agree |
| --- | --- | --- | --- | --- | --- | --- |

### PART 5. Demographics questions

What is your age? _________

How would you describe your gender?

- Male
- Female
- Non-binary / non-conforming
- Other (please specify) ______________
- Prefer not to say

## EXPERIMENT THREE

### PART 1. Demographics questions and test of existing belief on nuclear energy

Please answer the following questions about yourself:

1) What is your gender?

- Male
- Female
- Non-binary / non-conforming
- Other (please specify) ______________
- Prefer not to say

2) Are you currently a student? _____ No _____Yes

3) If you are a student, what is your major? _____________________________

4) What is your age? ___________

5) How many years of professional business experience do you have? ___________

6) Do you support the use of nuclear energy?

| 0  Not at all | 1 | 2 | 3 | 4 | 5 | 6 | 7  Completely |
| --- | --- | --- | --- | --- | --- | --- | --- |

### PART 2. Experimental manipulation

1. **Counterfactual generation condition:**

Please read the following story and then answer the question.

For some years after the arrival of Hastings as Great Britain’s Governor-General of India, the consolidation of power involved serious war. The first of these wars took place on the northern frontier of Bengal where the British were faced by plundering raids of the Gurkhas of Nepal. Attempts had been made to stop raids by an exchange of lands, but the Gurkhas would not give up their claims to a country under British control, and Hastings decided to deal with them once and for all. The campaign began in November, 1814. It was not glorious. The Gurkhas were only some 12,000 strong; but they were brave fighters, fighting in a territory well-suited to their raiding tactics. The older British commanders were used to war in the plains where the enemy would typically run away from a resolute attack. In the mountains of Nepal it was difficult to even find the enemy. The troops and transport animals suffered from the extremes of heat and cold, and the officers were slow to learn caution. The British overcame the Gurkhas and ultimately won the war.

Please list 2 thoughts about how this scenario might have turned out differently. That is, how could the Gurkhas have won this war?

1. __________________________________________________________
2. __________________________________________________________
3. **Control condition:**

Please read the following story and then answer the question.

For some years after the arrival of Hastings as Great Britain’s Governor-General of India, the consolidation of power involved serious war. The first of these wars took place on the northern frontier of Bengal where the British were faced by plundering raids of the Gurkhas of Nepal. Attempts had been made to stop raids by an exchange of lands, but the Gurkhas would not give up their claims to a country under British control, and Hastings decided to deal with them once and for all. The campaign began in November, 1814. It was not glorious. The Gurkhas were only some 12,000 strong; but they were brave fighters, fighting in a territory well-suited to their raiding tactics. The older British commanders were used to war in the plains where the enemy would typically run away from a resolute attack. In the mountains of Nepal it was difficult to even find the enemy. The troops and transport animals suffered from the extremes of heat and cold, and the officers were slow to learn caution. The British overcame the Gurkhas and ultimately won the war.

Please list 2 facts about this scenario.

1. __________________________________________________________
2. __________________________________________________________

### PART 3. Information disconfirming and confirming existing beliefs

Below are titles and brief descriptions for 6 current news articles about nuclear energy. Please examine the information about the articles and indicate how desirable it would be for you to read each article.

**Title - New Coolants Being Developed for Nuclear Reactors**

New liquid metal coolants may allow nuclear reactors to be smaller. The new coolants also reduce corrosion and the consumption of water for cooling. Significant additional research is needed to examine the long-term costs and benefits of liquid metal coolants.

Please indicate how desirable it would be for you to read the article described above:

| 1  Not at all desirable | 2 | 3 | 4 | 5 | 6 | 7 | 8 | 9 | 10 | 11  Extremely desirable |
| --- | --- | --- | --- | --- | --- | --- | --- | --- | --- | --- |

**Title - The Simple Argument for Keeping Nuclear Power Plants Open: We Need More Carbon-Free Power, not Less. *[Pro-nuclear information]***

By nearly all accounts, nuclear is [the most rapidly scalable form of carbon-free power](https://www.scientificamerican.com/article/the-world-really-could-go-nuclear/) invented. And, the technology is [rapidly improving](https://www.technologyreview.com/s/602051/fail-safe-nuclear-power/). Nuclear power plants emit no carbon pollution, no carbon monoxide, no sulphur oxides and no nitrogen oxides to the atmosphere.

Please indicate how desirable it would be for you to read the article described above:

| 1  Not at all desirable | 2 | 3 | 4 | 5 | 6 | 7 | 8 | 9 | 10 | 11  Extremely desirable |
| --- | --- | --- | --- | --- | --- | --- | --- | --- | --- | --- |

**Title - The World’s Nuclear Power Plants Pose a Grave Security Risk *[Anti-nuclear information]***

World leaders warn of nuclear power safety failings. Many countries are dependent on a source of energy where the slightest incident can have catastrophic consequences for millions of people and for tens of thousands of years. And these power plants face significant threats from terror attacks.

Please indicate how desirable it would be for you to read the article described above:

| 1  Not at all desirable | 2 | 3 | 4 | 5 | 6 | 7 | 8 | 9 | 10 | 11  Extremely desirable |
| --- | --- | --- | --- | --- | --- | --- | --- | --- | --- | --- |

**Title - Storage of Nuclear Waste Poses Major Threats to Health *[Anti-nuclear information]***

The energy debate must consider the lack of permanent repositories for dangerous nuclear waste. Spent fuel rods stored underground threaten countries with potential catastrophes. Governments have greatly underestimated the risks of nuclear waste fires triggered by events such as earthquakes or planned attacks.

Please indicate how desirable it would be for you to read the article described above:

| 1  Not at all desirable | 2 | 3 | 4 | 5 | 6 | 7 | 8 | 9 | 10 | 11  Extremely desirable |
| --- | --- | --- | --- | --- | --- | --- | --- | --- | --- | --- |

**Title - The World Needs Nuclear Energy for Electric Vehicles *[Pro-nuclear information]***

Global demand for electricity is predicted to grow 76% by 2030, and nuclear energy is set to play a key role in meeting the demand. Even a modest increase in the penetration of electric vehicles into the automotive market will require a significant increase in the development of nuclear energy.

Please indicate how desirable it would be for you to read the article described above:

| 1  Not at all desirable | 2 | 3 | 4 | 5 | 6 | 7 | 8 | 9 | 10 | 11  Extremely desirable |
| --- | --- | --- | --- | --- | --- | --- | --- | --- | --- | --- |

**Title – Nuclear Energy is Used to Power Deep Space Missions**

[Radioisotope thermoelectric generators](https://www.space.com/13702-nuclear-generators-rtg-power-nasa-planetary-probes-infographic.html) (RTGs) have been used to power spacecraft. These devices directly convert heat from decaying plutonium into electricity. The devices have no moving parts, making them ideal for applications in space.

Please indicate how desirable it would be for you to read the article described above:

| 1  Not at all desirable | 2 | 3 | 4 | 5 | 6 | 7 | 8 | 9 | 10 | 11  Extremely desirable |
| --- | --- | --- | --- | --- | --- | --- | --- | --- | --- | --- |

## EXPERIMENT FOUR

### PART 1. Test of misinformation beliefs about COVID vaccination safety:

Please indicate your level of agreement with each statement below: *(presented randomly)*

The ingredients in COVID-19 vaccines are dangerous.

| 1  Strongly disagree | 2 | 3 | 4 | 5 | 6 | 7  Strongly agree |
| --- | --- | --- | --- | --- | --- | --- |

Veganism is better for the environment compared to a diet including meat.

| 1  Strongly disagree | 2 | 3 | 4 | 5 | 6 | 7  Strongly agree |
| --- | --- | --- | --- | --- | --- | --- |

The natural immunity from being sick with COVID-19 is better than immunity from COVID-19 vaccination.

| 1  Strongly disagree | 2 | 3 | 4 | 5 | 6 | 7  Strongly agree |
| --- | --- | --- | --- | --- | --- | --- |

At any given time, humans only use about 10% of their brain's capacity.

| 1  Strongly disagree | 2 | 3 | 4 | 5 | 6 | 7  Strongly agree |
| --- | --- | --- | --- | --- | --- | --- |

*(Attention check 1)*

COVID-19 vaccines cause variants of the virus that causes COVID-19.

| 1  Strongly disagree | 2 | 3 | 4 | 5 | 6 | 7  Strongly agree |
| --- | --- | --- | --- | --- | --- | --- |

Thomas Edison invented the lightbulb.

| 1  Strongly disagree | 2 | 3 | 4 | 5 | 6 | 7  Strongly agree |
| --- | --- | --- | --- | --- | --- | --- |

COVID-19 vaccines can alter my DNA.

| 1  Strongly disagree | 2 | 3 | 4 | 5 | 6 | 7  Strongly agree |
| --- | --- | --- | --- | --- | --- | --- |

Drinking beverages high in caffeine leads to dehydration.

| 1  Strongly disagree | 2 | 3 | 4 | 5 | 6 | 7  Strongly agree |
| --- | --- | --- | --- | --- | --- | --- |

COVID-19 vaccines affect fertility.

| 1  Strongly disagree | 2 | 3 | 4 | 5 | 6 | 7  Strongly agree |
| --- | --- | --- | --- | --- | --- | --- |

### PART 2. Experimental manipulation

1. **Counterfactual generation condition:**

Please read the following story and then answer the question.

For some years after the arrival of Hastings as Great Britain’s Governor-General of India, the consolidation of power involved serious war. The first of these wars took place on the northern frontier of Bengal where the British were faced by plundering raids of the Gurkhas of Nepal. Attempts had been made to stop raids by an exchange of lands, but the Gurkhas would not give up their claims to a country under British control, and Hastings decided to deal with them once and for all. The campaign began in November, 1814. It was not glorious. The Gurkhas were only some 12,000 strong; but they were brave fighters, fighting in a territory well-suited to their raiding tactics. The older British commanders were used to war in the plains where the enemy would typically run away from a resolute attack. In the mountains of Nepal it was difficult to even find the enemy. The troops and transport animals suffered from the extremes of heat and cold, and the officers were slow to learn caution. The British overcame the Gurkhas and ultimately won the war.

Please list 2 thoughts about how this scenario might have turned out differently. That is, how could the Gurkhas have won this war?

1. __________________________________________________________
2. _________________________________________________________
3. **Control condition:**

Please list two random words.

1. __________________________________________________________
2. __________________________________________________________

PART 3. Facts contradicting false claims about COVID vaccination safety  *(randomly presented with fillers on Veganism, Brain Capacity, Thomas Edison, Caffeine)*

On the following pages, you will be provided with information that was collected from very reputable sources. It is important that you read all of the information because you will be asked to consider this information in the next section.

***Findings related to the Ingredients in COVID-19 Vaccines:***

- Nearly all the ingredients in COVID-19 vaccines are also ingredients in common foods, such as fats, sugars, and salts.
- COVID-19 vaccines do NOT contain any ingredients like preservatives, tissues (like aborted fetal cells), antibiotics, food proteins, medicines, latex, or metals.
- Exact vaccine ingredients vary by manufacturer.
- Pfizer-BioNTech and Moderna COVID-19 vaccines contain messenger RNA (mRNA) and the Johnson & Johnson/Janssen COVID-19 vaccine contains a harmless version of a virus that is unrelated to the virus that causes COVID-19.

***Findings related to Veganism:***

- Ranked by carbon emissions per 1,000 calories, tomatoes and broccoli rank higher (i.e. more emissions per calorie) than salmon, pork, chicken, tuna, milk, or eggs.
- Researchers modeled a scenario where Americans shifted from current diets to a diet with more fruits and vegetables as the USDA recommends; they found that this scenario would result in a 42% increase in energy use and a 16% increase in water use.
- A study modeled ten different diets to assess the carrying capacity of US agricultural land, and found that vegetarian diets that include dairy, or dairy plus eggs, lead to the highest carrying capacity for the US.
- Animals play an important role in nutrient cycling in agricultural systems, and allow us to capture energy and nutrients from perennial pasturelands, which would otherwise be wasted.

***Findings related to COVID-19 and Immunity:***

- Getting a COVID-19 vaccination is a safer and more dependable way to build immunity to COVID-19 than getting sick with COVID-19.
- Getting a COVID-19 vaccine gives most people a high level of protection against COVID-19 and can provide added protection for people who already had COVID-19.
- Studies have shown that those who do not get vaccinated after their recovery are more than 2 times as likely to get COVID-19 again than those who get fully vaccinated after their recovery.
- Getting sick with COVID-19 can offer some protection from future illness, but the level of protection from having COVID-19 varies depending on how mild or severe the illness was, the time since infection, and your age.
- Getting a COVID-19 vaccination is a safer way to build protection than getting sick with COVID-19 because getting sick with COVID-19 can cause severe illness or death, and it is difficult to predict who will have mild or severe illness.

*(Attention check 2)*

***Findings related to Brain Capacity:***

- All neurons have a resting firing rate, therefore they are never completely idle or unused.
- There is no part of the brain which is not active over the course of day.
- The process of natural selection generally does not yield biological adaptations that are not useful.
- The myth that humans use 10% of their brains is not supported by any neuroscientific evidence.

***Findings related to COVID-19 Vaccines and Fertility:***

- There has been no evidence that shows that any vaccines, including COVID-19 vaccines, cause fertility problems (problems trying to get pregnant) in women or men.
- Throughout the world, COVID-19 vaccination is recommended for people who are pregnant, trying to get pregnant now, or might become pregnant in the future, as well as their partners.
- Vaccination can also help pregnant women build antibodies that might protect their babies.

***Findings related to Thomas Edison:***

- Nearly 70 years before Edison, Humphrey Davy demonstrated an electric lamp to the Royal Society.
- In 1841, decades before Edison's work, a British inventor patented the enclosed bulb design to limit oxygen and keep the burners from catching fire.
- Edison hired a young physicist, Francis Upton, to help work on the light bulb. He had the breakthrough discovery that led to the light bulb development.
- Edison's patent for the electric light bulb was invalidated by the US Patent Office, who found that it was based on the work of another inventor.

***Findings related to COVID-19 Vaccines and Variants:***

- COVID-19 vaccines do not create or cause variants of the virus that causes COVID-19. Instead, COVID-19 vaccines can help prevent new variants from emerging.
- New variants of a virus happen because the virus that causes COVID-19 constantly changes through a natural ongoing process of change and mutation.
- As the virus spreads, it has more opportunities to change.
- High vaccination coverage in a population reduces the spread of the virus and helps prevent new variants from emerging.

***Findings related to Caffeine:***

- Drinking caffeine-containing beverages as part of a normal lifestyle doesn't cause fluid loss in excess of the volume ingested.
- Small increases in urine output, like those produced by mild diuretics, do not appreciably affect the hydration level of the body.
- A recent study found that the hydrating effects of caffeinated cola were not distinguishable from water.
- Research finds that in regular coffee drinkers, coffee consumption provides similar hydration to drinking water.

***Findings related to COVID-19 Vaccines and DNA:***

- COVID-19 vaccines do not change or interact with your DNA in any way.
- COVID-19 vaccines work by delivering instructions (genetic material) to our cells to start building protection against the virus that causes COVID-19.
- After the body produces an immune response, it discards all the vaccine ingredients just as it would discard any information that cells no longer need. This process is a part of normal body functioning.
- The genetic material delivered by mRNA vaccines never enters the nucleus of your cells, which is where your DNA is kept.
- Vaccines does not have the machinery needed to integrate affect our DNA, so they cannot alter our DNA.

### PART 4. Retest of misinformation beliefs about COVID vaccination safety:

Please indicate your level of agreement with each of the statements *(presented randomly and measured on a scale from 1 (strongly disagree) to 7 (strongly agree)*:

The ingredients in COVID-19 vaccines are dangerous.

Veganism is better for the environment compared to a diet including meat.

The natural immunity from being sick with COVID-19 is better than immunity from COVID-19 vaccination.

At any given time, humans only use about 10% of their brain's capacity.

COVID-19 vaccines cause variants of the virus that causes COVID-19.

Thomas Edison invented the lightbulb.

COVID-19 vaccines can alter my DNA.

Drinking beverages high in caffeine leads to dehydration.

COVID-19 vaccines affect fertility.

### PART 5. Demographics questions

What is your age? _________

How would you describe your gender?

- Male
- Female
- Non-binary / non-conforming
- Other (please specify) ______________
- Prefer not to say

## **Table S3.** Experiment 4 – Tests for Potential Effects of Country of Origin and Socio-Economic Status on Changes to Misinformation Beliefs related to COVID Vaccination

| **Covariate** | **Dependent Variable** | **F-statistic** | **p-value** |
| --- | --- | --- | --- |
| Socio-Economic Status | Dangerous | 1.06 | 0.39 |
|  | Immunity | 1.48 | 0.15 |
|  | Variants | 1.02 | 0.42 |
|  | Fertility | 1.13 | 0.34 |
|  | DNA | 0.56 | 0.83 |
| Country | Dangerous | 1.20 | 0.27 |
|  | Immunity | 0.12 | 0.73 |
|  | Variants | 0.11 | 0.74 |
|  | Fertility | 1.41 | 0.24 |
|  | DNA | 0.04 | 0.84 |

Tests are based on ANCOVA models with the counterfactual treatment condition as the independent variable and changes in the five misinformation beliefs related to COVID vaccination as the dependent measures. Covariates for socio-economic status and country of origin were included one at a time. Results for all measures of changes in misinformation beliefs are consistent across individuals from the United States and United Kingdom, and results are consistent for individuals of higher and lower socio-economic status.
